# Development of a Model of Hemispheric Hypodensity (“Big Black Brain”)

**DOI:** 10.1089/neu.2018.5736

**Published:** 2019-02-09

**Authors:** Beth A. Costine-Bartell, Declan McGuone, George Price, Eleanor Crawford, Kristen L. Keeley, Jennifer Munoz-Pareja, Carter P. Dodge, Kevin Staley, Ann-Christine Duhaime

**Affiliations:** ^1^ Department of Neurosurgery, Massachusetts General Hospital, Boston, Massachusetts.; ^2^ Department of Neurosurgery, Harvard Medical School, Boston, Massachusetts.; ^3^ Department of Pathology, Yale School of Medicine, New Haven, Connecticut.; ^4^ Department of Pediatrics, University of Florida Health Shands Children's Hospital, University of Florida College of Medicine, Gainesville, Florida.; ^5^ Department of Anesthesiology, Children's Hospital at Dartmouth, Dartmouth Medical School, Lebanon, New Hampshire.; ^6^ Departments of Neurology, Massachusetts General Hospital and Harvard Medical School, Boston, Massachusetts.

**Keywords:** abusive head trauma, apnea, gyrencephalic, seizures, subdural hemorrhage

## Abstract

Subdural hematoma (SDH) is the most common finding after abusive head trauma (AHT). Hemispheric hypodensity (HH) is a radiological indicator of severe brain damage that encompasses multiple vascular territories, and may develop in the hemisphere(s) underlying the SDH. In some instances where the SDH is predominantly unilateral, the widespread damage is unilateral underlying the SDH. To date, no animal model has successfully replicated this pattern of injury. We combined escalating severities of the injuries and insults commonly associated with HH including SDH, impact, mass effect, seizures, apnea, and hypoventilation to create an experimental model of HH in piglets aged 1 week (comparable to human infants) to 1 month (comparable to human toddlers). Unilateral HH evolved over 24 h when kainic acid was applied ipsilateral to the SDH to induce seizures. Pathological examination revealed a hypoxic-ischemic injury-type pattern with vasogenic edema through much of the cortical ribbon with relative sparing of deep gray matter. The percentage of the hemisphere that was damaged was greater on the ipsilateral versus contralateral side and was positively correlated with SDH area and estimated seizure duration. Further studies are needed to parse out the pathophysiology of this injury and to determine if multiple injuries and insults act synergistically to induce a metabolic mismatch or if the mechanism of trauma induces severe seizures that drive this distinctive pattern of injury.

## Introduction

Traumatic brain injury (TBI) is the leading cause of acquired death and disability in children, and in children under age 2, the majority of severe TBI is due to abusive head trauma (AHT).^1^ Acute subdural hemorrhage (or hematoma; SDH) is the most common intracranial abnormality resulting from AHT and is observed radiologically in 81–91% of cases.^2–4^ Hemispheric hypodensity (HH) is an injury pattern observed radiologically that is unique to infants and toddlers where there is uniform loss of gray-white differentiation in the entire hemisphere associated with the SDH encompassing all three vascular territories despite patent, large vessels.^3–5^ The HH pattern may be visible by imaging within hours of arrival to the hospital or may evolve over days.^6^ Significant biomechanical forces result in SDH, subarachnoid hemorrhage, often with evidence of an impact event (skull fracture, scalp contusions, or subgaleal hematomas), and apnea.^7^ Eventual atrophy of the entire cortical ribbon in the hemisphere underlying the SDH may result in a lifetime of severe motor impairment (hemiparesis or quadraparesis), cognitive impairment, post-traumatic epilepsy, and blindness, or may result in death.^6,8,9^ Unilateral HH is a striking pattern observed in 22% of cases where the SDH is restricted to one hemisphere, mass effect is present, and only the hemisphere underlying the SDH is hypodense on CT scan.^4,5^ The frequency of HH (hemispheric bilateral or hemispheric unilateral) is reported to develop in 25–50% of AHT cases presenting to the hospital.^2–4,9^ Mortality rates are double in children with AHT who develop HH versus no development of HH (46 vs. 18%^3^; 25 vs. 1%).^4^ Radiological findings of HH may be subsequently obscured on pathological examination by intervening evolution of TBI, including global anoxic encephalopathy, and therefore is not observed by the time autopsy examination of the brain is performed. As is the case with most AHT, unilateral HH is not always fatal.^6^ At autopsy, there is usually evidence of an impact event, a hypoxic-ischemic pattern, and/or traumatic axonal injury.^10–13^ The areas of injury in HH may be referred to by various clinicians as “stroke,” “edema,” “infarct,” or “hypoxic-ischemic injury” via imaging or at autopsy, but do not indicate the responsible pathological mechanisms.^4,10,11^ Involvement of multiple vascular territories, lack of evidence of restricted arterial blood flow, and cases of unilateral HH indicate the pathophysiological cascades leading to a mismatch of metabolic demand, and substrate delivery can occur in the absence of occluded blood vessels or global hypoxia/anoxia.^5^ It should be noted that HH has been documented to occur from accidental TBI and the presence of HH does not indicate mechanism or manner of injury.^5^ In infants, in whom seizures are common and often subclinical, electrophysiological disturbances may contribute to the pathophysiological cascades.^9^ Infants and toddlers who develop HH often are noted to have periods of apnea; however, it is unlikely that this injury is purely driven by anoxia given the instances of unilateral distribution of HH.^3,5^ The pathophysiological mechanisms damaging the cerebral cortex under an SDH with relative sparing of the contralateral hemisphere, in the context of global apnea, remain unknown. No known interventions prevent the development of secondary parenchymal damage within the affected hemisphere after AHT in children.^3^

A significant barrier to understanding the pathophysiology in AHT to date is the lack of an animal model that replicates HH. Little progress has been made in the 2 decades since the first longitudinal imaging studies described age-dependent differences in AHT injuries in children.^2,9^ Models comprising SDH alone do not result in the extensive tissue damage pattern that occurs after AHT, including HH.^14–17^ SDH causes localized edema, decreased tissue perfusion, and excitotoxicity restricted to the tissue adjacent to the SDH.^16–18^ Although the exact etiological mechanisms that cause injury in children with AHT remain controversial, repetitive motions attempting to model aspects of shaking have been employed in some animal models to investigate injury, but none has resulted in the extensive SDH nor the widespread hypoxic-ischemic type damage observed after the more severe forms of AHT in children.^19–21^

Here we present a unique, immature, large-animal model designed to replicate the constellation of injuries and insults typical in AHT-associated patterns of predominantly unilateral hypodensity with patterns of tissue damage seen in children. We used our well-characterized cortical impact model to induce mechanical trauma, as children with HH often have evidence of blunt mechanical trauma of some type.^6,22,23^ Our cortical impact is reproducible and injures both gray and white matter.^24^ We also induced mass effect, apnea, hypoventilation, and seizures to assess the role of these insults. No other model of AHT has induced or directly investigated seizures to determine if seizures are part of the pathophysiological cascade of HH.^6,7^ The goal of this model of AHT is to replicate the potentially synergistic multi-factorial injury cascades within comparable developmental stages and brain morphology of human infants and toddlers. Identification of key components that lead to extensive tissue damage after AHT, such as seizures, and their potential effect on distribution of damage may produce targets for future therapies.

## Methods

### Protocols general to all experimental phases

The experimental optimization of this model was carried out in three phases. The purpose of Experimental Phase 1 was to test the implementation of the insults and the survivability of the combinations of injuries and insults in escalating doses. The purpose of Experimental Phase 2 was to determine the effect of potentially synergistic combinations of sub-lethal injuries and insults on the pattern of brain pathology. The purpose of Experimental Phase 3 was to further refine the model to restrict damage to the hemisphere under the SDH with the injuries and insults scaled to age to develop a model that can test the effects of age on injury pattern in future experiments. Phases are described in more detail in Table 1 and in subtitled sections below.

**Table 1.** Details of Experimental Injuries and Insults

*Parameter*	*Experimental phase 1*	*Experimental phase 2*	*Experimental phase 3*
*Age*	*PND 7 or 21*	*PND 21*	*PND 30*
Cortical impact	Scaled to displace 1% brain volume PND 7 = 1.04 cm in diameter and indenting to a depth of 4.8 mm PND 21 = 1.07 cm in diameter with 5.9-mm indention	Scaled to displace 1% brain volume: 1.07 cm in diameter with 5.9-mm indention	Scaled to displace 1% brain volume: 1.07 cm in diameter with 5.9-mm indention
Mass effect balloon	No balloon or 1–3 mL (2.4–7.2% of estimated brain weight); 15 min	1–3 mL (2.4–7.2% of estimated brain weight); 15 min	Scaled to 5% of estimated brain weight: PND 30 = 2.7 mL 15 min rostral +15 min caudal = 30 min total
Subdural hematoma	2–5 mL (4.8–12% of estimated brain weight) with or without epidural blood (3 mL, 7.2%)	1.5–4.5 mL (3.6–10.9% of estimated brain weight; average = 2.8 ± 0.11).	Scaled to 10% of estimated brain volume: PND 30 = 5.2 mL blood
Seizure permissive anesthesia	Methohexital (1.5 mg/kg bolus, 50–150 μg/kg/min infusion)	Methohexital (1.5 mg/kg bolus, 50–150 μg/kg/min infusion)	Dexmedetomidine (20 μg/kg/h), morphine (3.5 mg/kg/h), chlorpromazine (2 mg/kg bolus)
Epileptic agent	2.0–2.5 mg/kg bicuculline (IV)	2.5 mg/kg bicuculline (IV)	Kainic acid (42 μg/kg); intraparenchymal or mixed with the subdural blood
Apnea	2 rounds of 2–3 min of apnea	2 rounds of 3 min of apnea	1 round of 1 min of apnea
Hypoventilation	2 rounds of 5–10 min	2 rounds of 10 min	1 round of 10 min
Time recovered	None	None	0–12 h
Survival duration	10 h	9 h	24–25 h
Early death	1 of 4; 25%	4 of 20; 20%	1 of 12; 8%

**FIG. 1.**
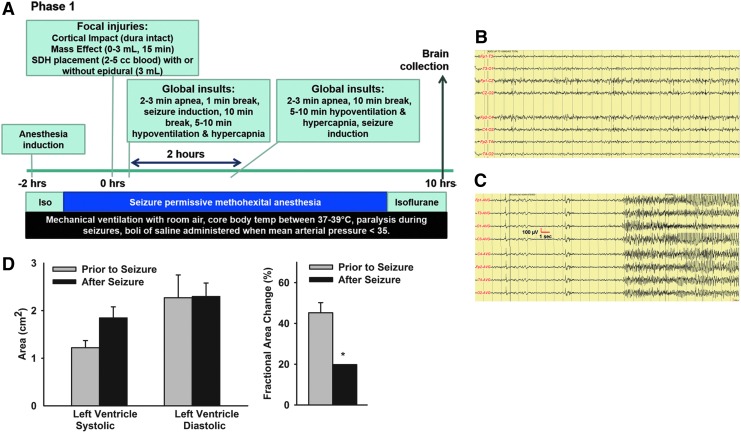
Experimental Phase 1 of model development. **(A)** Schematic of experimental time line and procedures. **(B)** PTZ failed to induce seizures with methohexital anesthesia. **(C)** Bicuculline (intravenously) rapidly induced seizures. **(D)** After seizures, induced with bicuculline piglets had decreased left ventricular contractility and a lower fractional area change (^*^means ± SEM differ, *p* < 0.05) indicating reduced ejection fraction and at least temporary myocardial dysfunction. PTZ, pentylenetetrazol; SEM, standard error of the mean.

**FIG. 2.**
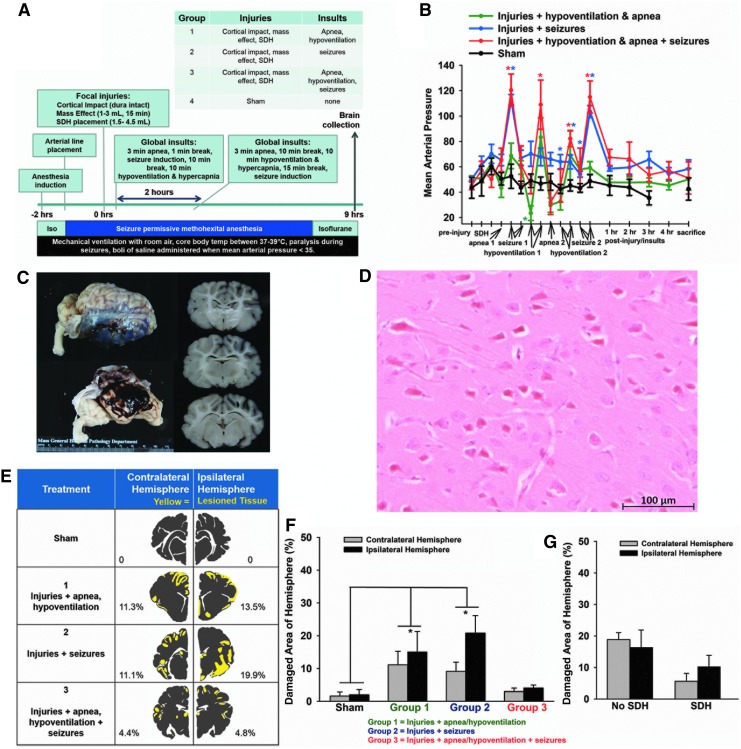
Experimental Phase 2 of model development. **(A)** Schematic of experimental time line and procedures. **(B)** Mean arterial pressure increased after seizure in groups with seizure and declined during apnea and hypoventilation in groups with apnea and hypoventilation (^*^means ± SEM differ, *p* < 0.05). **(C)** Photograph of the brain with a subdural hematoma (SDH) and dusky gray appearance of the cortical ribbon underlying the SDH. **(D)** H&E-stained tissue section demonstrating the widespread “red neurons” primarily observed in Experimental Phase 2 of model development (scale bar = 100 μM) depicting typical red neurons with cell shrinkage, cytoplasmic acidophilia, and loss of nuclear detail. **(E)** Representative distribution of tissue damage in each group (evaluated using H&E staining) demonstrated bilateral, patchy, widespread, predominantly cortical injury (yellow = damage, gray = healthy tissue). **(F)** The distribution of tissue damage did not differ ipsilateral versus contralateral to the focal injuries in all groups, but was bilaterally greater in Group 1 and 2 than in sham piglets (*p* < 0.05). **(G)** Eight injured piglets failed to have an SDH, but had a subarachnoid hemorrhage instead. The presence of absence of an SDH did not affect the pattern of red cell change in the hemisphere ipsilateral to the focal injuries or contralateral. H&E, hematoxylin and eosin; SEM, standard error of the mean.

**FIG. 3.**
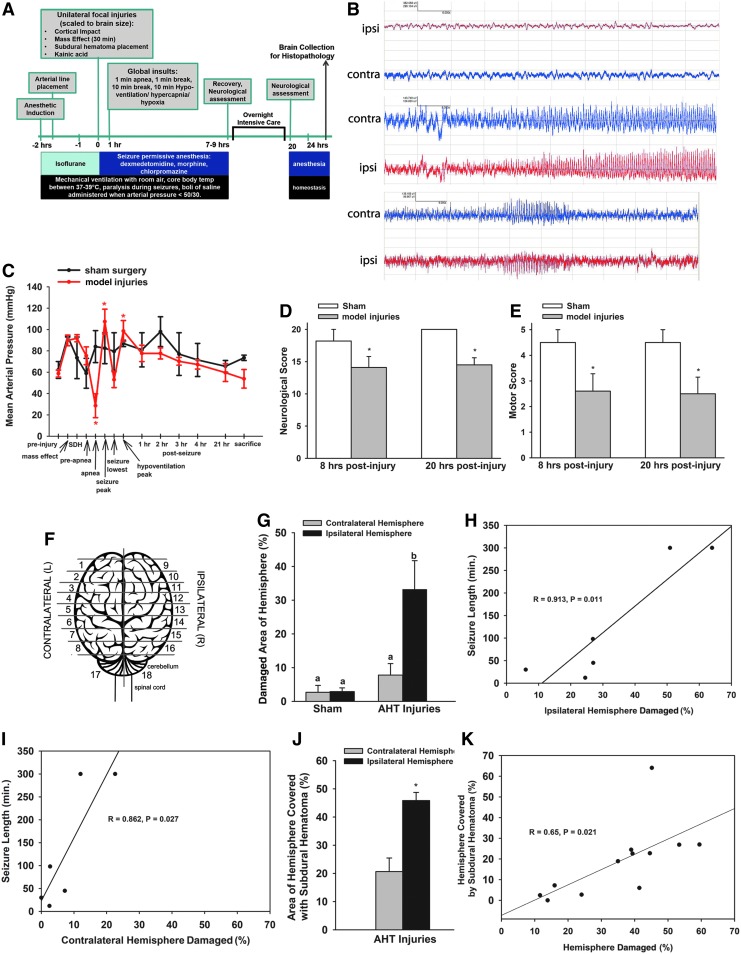
Experimental Phase 3 of model development. **(A)** Schematic of experimental time line and procedures. **(B)** EEG of two- channel bipolar montage on the hemisphere ipsilateral (red) or contralateral (blue) to the focal injuries and subdural hematoma (SDH) before kainic acid (top panel), after kainic acid (middle panel), and 3 h post-kainic acid. **(C)** In piglets receiving model injuries, mean arterial pressure (MAP) changed over time, but was constant in sham piglets (interaction, *p* = 0.02). MAP was lower during apnea compared with pre-apnea (*p* = 0.006), and was elevated during seizures (*p* = 0.003) and hypoventilation (*p* = 0.041) compared with pre-injury. Model injuries reduced neurological **(D)** and motor scores **(E)** compared with sham piglets. **(F)** Schematic indicating the sampling from 5-mm blocks rostral to caudal to estimate volume of tissue damage throughout the cerebrum. **(G)** Tissue damage was greater in the hemisphere ipsilateral to the SDH and blunt impact versus contralateral. The percentage of the hemisphere that was damaged was positively correlated to total estimated seizure duration in both the ipsilateral **(H)** and contralateral **(I)** hemispheres. **(J)** SDH covered 50% of the ipsilateral hemisphere, and 20% of the contralateral hemisphere (most commonly located along the parasagittal surface). **(K)** The percentage of damaged cerebral hemisphere positively correlated with the percentage of the hemisphere covered by SDH. EEG, electroencephalogram.

See Figs. 1–3 for the depiction of timing.

IV, intravenous; PND, post-natal day.

#### Surgery and brain collection

Male, Yorkshire piglets (Earle Parsons & Sons, Inc., Hadley, MA) were housed and fed as previously described after arrival at post-natal day (PND) 5, 19, or 28.^25^ Milk replacer (PND 5) or grain pellets (PND 19 and 28) were removed the evening prior to surgery and piglets were given a liquid electrolyte solution (BlueLite; TechMix, Inc., Stewart, MN). A total of 48 piglets (Experimental Phase 1, *n* = 8; Experimental Phase 2, *n* = 25; Experimental Phase 3, *n* = 15) were used to develop this model in three experimental phases. Injuries were scaled to PND 7, similar to human infants, and PND 21–30, which are similar to human toddlers (described in detail previously).^22,26^ All protocols and procedures were in accordance with the guidelines of the American Veterinary Association and the National Institutes of Health, and were approved by the Animal Care and Use Committee at Dartmouth College or Massachusetts General Hospital.

Consistent with the severe nature of the injury being modeled, there were 5 deaths prior to the end of the experiment (Experimental Phase 1 = 1 death; Experimental Phase 2 = 4 deaths). Four deaths were due to cardiac arrest and failed resuscitation, and 1 death was likely due to an adverse reaction to anesthesia during transition between anesthetic regimens (Experimental Phase 2).

General anesthesia was induced with 5% isoflurane delivered via nose-cone mask, and an intravenous (IV) catheter was placed in a limb or ear vein. End-tidal CO_2_, oxygen saturation, blood pressure, heart rate, and core body temperature were monitored and recorded. Core body temperature as measured via a rectal probe was maintained between 37 and 39°C utilizing a heated pad and Bair Hugger forced air blanket. Buprenorphine (0.002 mg/kg for PND 7 piglets and 0.02 mg/kg for PND 21–30 piglets; intramuscularly [IM] or IV) was administered prior to incision. Piglets were intubated and mechanically ventilated with room air and minute ventilation was adjusted to maintain an end-tidal CO_2_ between 35 and 45 mm Hg except during episodes of induced apnea and hypoventilation (described below). Saline boluses (5–10 mL/kg) were administered for hypotension (mean arterial pressure [MAP] <35 mm Hg). If saline was not successful in increasing blood pressure, then epinephrine (0.005 mg/kg) or ephedrine (0.05 mg/kg) was given via IV. If cardiac arrest occurred, then chest compressions were administered. Before skin incisions, bupivacaine was administered subcutaneously (maximum total = 2.5 mg/kg). Venous or arterial blood was assessed with an iSTAT-handheld® (Abbott Point of Care, Princeton, NJ) at regular intervals (described in detail below for each experimental phase), and dextrose was administered (0.75 mg/kg, IV) if the piglet was hypoglycemic (glucose <70 mg/dL).

An arterial line was placed in the femoral artery as peripheral blood pressure could be undetectable via blood-pressure cuff due to injuries/insults and potentially from anesthetics (Experimental Phases 2 and 3), and was used for blood collection for iSTAT analysis, for a blood source for subdural placement, and for future biomarker analysis. A central venous catheter was placed in either the femoral vein or external jugular vein for administration of medications (Experimental Phase 3). No more than 15% of estimated blood volume was collected over the course of the experiment.

To model the frequent concomitant physiological events seen in children with severe AHT, which include hypoventilation and/or apnea and seizures, piglets received focal injuries (cortical impact, SDH, and mass effect) and global insults (apnea, hypoventilation, and seizures) in patterns that evolved over the three experimental phases of the experiment (Figs. 1A, 2A, 3A). For creation of focal injuries, the scalp was incised, and a burr hole (2 cm in diameter) was made through the skull at the mid-portion of the right coronal suture. With the dura closed, a cortical impact was deployed as previously described (Table 1).^22^ After the cortical impact, subjects received an injected autologous SDH and epidural balloon inflation to create additional mass effect with volumes and durations as described below (Table 1). For the SDH, the burr hole was enlarged medially and an angiocatheter (24 gauge) was inserted into the dura as previously described.^27^ Leak of cerebrospinal fluid (CSF) through the catheter indicated placement in the subarachnoid space, in which case the catheter was removed and replaced within the subdural space as confirmed by no CSF egress. Autologous blood (1.5–5.2 mL; 3.6–12% of estimated brain weight; Table 1) was introduced through the angiocatheter slowly over 5 min, creating an SDH; the blood was allowed to clot, and the catheter was removed. Additional temporary mass effect with the potential for midline shift and increased intracranial pressure was achieved by introduction of a Fogarty embolism catheter (size 6 French [6F]) into the epidural space and was inflated slowly (1–3 mL; 2.4–7.2%; Table 1) over 1 min and left in place for 15–30 min (Table 1). Seizure-permissive anesthesia was initiated (described below for each experimental phase) and isoflurane was discontinued. When end-tidal isoflurane reached zero, with the plane of anesthesia sufficient (unresponsiveness to the pinch reflex and lack of muscle movement), neuromuscular blockade was initiated (0.8 mg/kg rocuronium) before seizure induction to prevent de-instrumentation with convulsions and breathing during apnea and hypoventilation. Rounds of apnea and hypoventilation were administered as described for each experiment below (Table 1). Apnea (1–3 min) was achieved by turning off the ventilator and clamping the endotracheal tube, and hypoventilation was achieved by turning off the ventilator and manually ventilating the piglet to ∼4 breaths per min on room air or supplemented with oxygen to allow desaturation of oxygen to 80–90% and hypercarbia with the goal of reaching an end-tidal CO_2_ of ∼60 mm Hg.

Piglets were maintained on anesthesia (Experimental Phases 1 and 2) or recovered (Experimental Phase 3) to allow evolution of the pathophysiological cascades that might induce HH. Brains were collected 9–25 h after the cortical impact under deep anesthesia with isoflurane (Table 1).

#### Hematoxylin and eosin (H&E) staining

Sections were placed on a 60°C heat block for 10 min, then rehydrated with xylene (twice for 5 min), 100% ethanol (twice for 1 min), 95% ethanol (twice for 1 min), and 70% ethanol (once for 1 min). Between all subsequent steps, sections were washed 3 times in de-ionized H_2_O (dH_2_O). Sections were stained with Gill's III Hematoxylin for 2 min (Thermo Fisher Scientific, Waltham, MA), 1% acid alcohol for three washes, bluing reagent (Thermo Fisher Scientific) for 30 sec, and 1% alcoholic Eosin Y (Electron Microscopy Sciences; pH 4.5) for 30 sec. Slides were then dehydrated with 70% ethanol (once for 1 min), 95% ethanol (twice for 1 min), 100% ethanol (twice for 1 min), and xylene (twice for 5 min). Permount (Fisher Scientific, Fair Lawn, NJ) was applied to slides and coverslipped.

#### Immunohistochemistry for active caspase-3, APP, and albumin

To determine the presence of apoptosis, axonal damage, and vasogenic edema, sections were de-paraffinized and rehydrated as described above and probed for active caspase-3, APP, or albumin, respectively (see Table 2 for antibody details). Sections were treated with 3% hydrogen peroxide (Medline, Mundelein, IL) for 15 min to quench endogenous peroxide activity (Table 2). Slides were placed in pre-heated buffer (Table 2) and heated for 120°C for 10 min and 90°C for 20 min in a pressure cooker (DAKO, Carpinteria, CA). After 10 min of equilibration to room temperature, sections were rinsed in phosphate-buffered saline (137 mM NaCl, 2.7 mM KCl, 10 mM phosphate buffer (EMD Chemicals, Inc., Gibbstown, NJ; pH 8.0) with 1% Tween 20 (PBST; Sigma-Aldrich, St. Louis, MO) for 5 min to increase permeability followed by a dH_2_O rinse for 1 min. Slides for caspase-3 staining were washed 3 times for 5 min each in dH_2_O without the PBST step. A PAP pen (Life Technologies, Frederick, MD) was used to create a hydrophobic barrier surrounding the tissue, and slides were incubated with a blocking solution (Table 2). For the cleaved caspase-3 SignalStain Antibody diluent and SignalStain Boost Detection Reagent (Cell Signaling Technology, Danvers, MA) were used. The albumin and APP protocols used undiluted normal horse serum in Super Sensitive Wash Buffer (wash buffer; BioGenex, Fremont, CA) for blocking and the Vectastain Universal Elite ABC Kit PK-6200 (Vector Laboratories, Burlingame, CA) according to manufacturer's instructions. Slides were incubated overnight at 4°C with the primary antibody diluted in the blocking buffer (Table 2). For negative controls, the primary antibody was omitted. Slides were washed 3 times for 8 min each in PBST before application of the secondary antibody and color reagent according to manufacturer's instructions (Table 2).

**Table 2.** Immunohistochemistry Details

	*Quenching*	*Antigen retrieval buffer*	*Blocking*	*Primary antibody*	*Secondary antibody*	*Color reaction*
Albumin	3% H_2_O_2_ 15 min, 5 min dH_2_O rinse	1 M Tris 0.5 M EDTA buffer (pH = 8.0)	Normal horse serum in Optimax buffer, 30 min	Rabbit anti-human Ab137885 1:250 (Abcam, Cambridge, MA)	Vectastain Universal Elite ABC Kit	DAB (Vector Labs) solution 3 min
APP	3% H_2_O_2_ 15 min, 5 min dH_2_O rinse	1 M Tris 0.5 M EDTA buffer (pH = 8.0)	Normal horse serum in Optimax buffer, 30 min	Mouse anti-human MAB348 1:100 (Millipore, Billerica, MA)	Vectastain Universal Elite ABC Kit	DAB solution 1–3 min
Active-caspase-3	3% H_2_O_2_ for 10 min during deparaffin/hydration	10 mM sodium citrate buffer (pH = 7.5)	5% normal goat serum in SignalStain® Antibody Diluent #8112	Rabbit anti-human Ab2302 1:500 (Abcam)	SignalStain Cleaved Caspase Kit (12692S)	DAB solution 6 min

Slides were counterstained with hematoxylin, dehydrated following the rehydration process in reverse as described above (see H&E section), then mounted with Shandon Mount (Thermo Fisher Scientific, Cheshire, United Kingdom) and coverslipped. After 30 min, nail polish (Electron Microscopy Sciences, Hatfield, PA) was applied to the border of each coverslip to seal the slide.

#### Analysis of tissue damage and APP staining pattern

Sections used for analysis were photographed (Rebel, Cannon USA, Melville, NY) with a ruler in the frame. These photographs were printed and the area of brain damage (H&E stained sections) or pattern of APP or albumin staining was observed using the 5 × and 10 × objectives and drawn on the paper map of the slide using specific landmarks on the slide and slide photograph.^28–30^

An area was determined to be “damaged” if the tissue had (1) “red neurons,” (2) acute tissue hemorrhage, or any of the following changes in combination with or adjacent to areas of red neurons: (3) vacuolization around blood vessels, or (4) vacuolization of neuropil, or (5) vacuolization around neurons. Operational criteria to classify neurons as red neurons were the presence of all three of the following features: (1) cell shrinkage with perineuronal vacuolization, (2) cytoplasmic hypereosinophilia, and (3) nuclear pyknosis. Vacuolization alone was not included in the calculation of brain damage areas. Scientists (BB, GP) trained and audited by a forensic neuropathologist (DM) delineated the areas of damage. The sections chosen for analysis differed per experimental phase (noted below). In addition, we qualitatively examined APP, caspase, and albumin staining patterns.

The maps of damage identified via H&E were scanned and Adobe Photoshop CS5 (San Jose, CA) was used to determine the area of damage in each hemisphere. Hemispheres were analyzed separately. Details on determining damage areas and volume are described below in the protocol-specific sections of Experimental Phases 2 and 3.

### Protocol specific to Experimental Phase 1

A total of 8 piglets aged 7 (*n* = 3; 2.3 ± 0.1 kg) or 21 days (*n* = 5; 5.6 ± 0.4 kg) were used. Piglets were treated as above with the following exceptions. Blood pressure was monitored with a blood-pressure cuff that had a lower detection limit of 19 mm Hg for MAP.

Four piglets (PND 7 or 21) were used for subdural placement optimization and testing of epileptic agents with the seizure-permissive anesthesia protocol. Isoflurane was withdrawn and a bolus of 1.5 mg/kg methohexital (Brevital® Sodium; JHP Pharmaceuticals, Parsippany, NJ) was administered, followed by an infusion of 50–150 μg/kg/min that was titrated to normal vital signs. To eliminate motor expression of seizures and allow physiological monitoring, neuromuscular blockade was induced with rocuronium (0.8 mg/kg per 10 min) via an IV catheter titrated to less than 2 twitches by Train-of-Four monitoring. Electroencephalograms (EEGs) were obtained via a montage consisting of scalp-applied electrodes in two longitudinal bipolar configurations on the left and on the right with adjustments made for the craniectomy using silver-coated disc electrodes (1 cm; Grass Technologies, West Warwick, RI) and a Comet Grass-Telefactor EEG machine (Grass Technologies). Results were reviewed by a board-certified pediatric neurologist. Pentylenetetrazol (50–150 mg/kg) and penicillin applied to the surface of the cortex, and bicuculline, a competitive γ-aminobutyric acid A (GABA_A_) receptor antagonist, were tested as epileptic agents in PND 7 piglets. Bicuculline was prepared by dissolving in 0.1 N HCL, and adding 0.1 N NaOH until pH = 4.5, resulting in a final concentration of 1.4–1.6 mg/mL.

Four piglets (PND 21) were used to test escalating combinations of injuries and insults starting with low volumes of subdural blood, short-duration apnea, and short-duration hypoventilation and then increasing the volume and duration of insults with the addition of mass effect (Table 1). After injuries (cortical impact, SDH with or without mass effect), insults were clustered together with apnea induction, a 1-min break, seizure induction, and then a 10-min break followed by hypoventilation induction. A second round of insults 2 h later started with apnea, a 2-min break, then hypoventilation, followed by seizure induction. Cerebral oxygen saturation of the brain was measured via near-infrared spectroscopy as pulse oximetry units attached peripherally failed during apnea. Poor blood flow to the skin was observed following seizure indicating impaired cardiac function. An echocardiogram measuring two-dimensional systolic area, diastolic area, and stroke area was obtained prior to and after seizure induction in two subjects by a cardiologist or cardiac scientist. The ratio of diastolic area to stroke area was used to calculate the fractional area change. Arterial blood was analyzed with an iSTAT in one subject prior to injuries and insults and at the end of the experiment. At the end of the experiment, under anesthesia, piglets were euthanized by IV or intracardiac administration of Euthasol® (Vibrac Animal Health, Fort Worth, TX). The skull was opened to confirm the placement of an SDH. One piglet died before the end of the experiment, 2 h after the end of the second round of insults due to cardiac arrest and failed resuscitation.

### Protocol specific to Experimental Phase 2

A total of 25 male PND 21 piglets (6.7 ± 0.25 kg) were used. Piglets were assigned to four treatments: injuries + apnea with hypoventilation, injuries + seizures, injuries + apnea with hypoventilation + seizures, or sham injuries (Fig. 2) with the parameters as described in Table 1. A subset of piglets received an echocardiogram to assess cardiac function (*n* = 7). A total of 4 animals died before the end of the experiment. Three animals died from cardiac arrest, and all were in a treatment group including apnea. One animal, which was in the seizure only group, likely died from an adverse anesthetic event in the transition from seizure permissive protocol to isoflurane. One animal (injuries + seizures group) was excluded from damage area assessment due to the presence of an intraparenchymal hemorrhage in the cortex. Arterial or venous blood was collected for blood gases and metabolites prior to injury, 30 min to 7 h post-injury, and prior to sacrifice. If venous blood was collected, values were adjusted for pH (+0.03), HCO_3_ (−1 meq/L), and PCO_2_ (−5 mm Hg). Seizures induced by IV bicuculline were estimated via a heart rate over 250 bpm based on correlation between this degree of tachycardia and seizures on EEG recordings from Experimental Phase 1 and were not directly recorded in Experimental Phase 2.

At the end of the experiment, piglets were deeply anesthetized with 3–5% isoflurane and euthanized via exsanguination by transcardial perfusion with 0.9% saline followed by 10% phosphate buffered formalin as previously described.^23,25^ The brain was removed leaving the ventral dura intact and post-fixed at 4°C for up to 1 month. The eyes with attached optic nerves were removed by dissection through the floor of the anterior cranial fossa into the orbital space to expose the globe and contents of the orbit in a subset of injured piglets (*n* = 3). The eyes were then formalin fixed, photographed, examined grossly for presence of optic nerve sheath hemorrhage, and sectioned horizontally from the posterior pole to the corneal rim, preserving the optic nerve. The eyes were examined macroscopically for retinal hemorrhage, and then were whole mount paraffin embedded. Tissue sections were examined for microscopic retinal hemorrhage using H&E staining. Brains were weighed, photographed with and without the dura, and the presence of subdural blood and/or subarachnoid blood was noted. Whole brains were photographed and sectioned in the coronal plane into 5-mm blocks starting at the anterior edge of the rostral gyrus. Blocks were paraffin embedded and stored in sealed containers at room temperature. Three 5-mm blocks at the level of the rostral gyrus (Fig. 3F; blocks corresponding to 3/11, 4/12, and 5/13) were serially sectioned into 10-μm sections, and every other section was mounted on poly-L-lysine-coated glass slides (Plain Micro Slides, Corning, Inc., Corning, NY), dried, and stored in labeled slide boxes at room temperature.

H&E-stained sections were qualitatively assessed for mechanical injury (contusion area, presence of necrosis, extravasated red blood cells, tissue tears, and axonal swelling), as well as for red neurons. Apart from focal blunt impact injury, no other lateralizing pattern of injury was observed: red neurons were widespread throughout the cerebrum, predominantly at watershed areas (see Results section for details); therefore, two sections containing both hemispheres (one section from two blocks: 4/12 and 5/13; Fig. 3F) were mapped for tissue damage as described above. The area of tissue damage was taken as a percentage of the area of the entire hemisphere and averaged among sections per hemisphere per piglet.

### Protocol specific to Experimental Phase 3

A total of 14 piglets aged 30 days (9.2 ± 0.36 kg) were used. Piglets were treated as described in the general methods with the following exceptions. Piglets received Cetacain® spray (Cetylite Inc., Pennsauken, NJ) prior to intubation. Piglets received 25 mg/kg cefazolin via IV after IV placement. CSF was collected via 23-gauge catheter from the subarachnoid space in the ventral portion of the burr hole of the skull (200–400 μL) or via the cisterna magna (0.5–1.5 mL) with an 18-gauge spinal needle prior to injury and prior to sacrifice for future analysis.

To facilitate the determination of the effect of age on the pathophysiology of this injury in planned future experiments, the GABA_A_ receptor was avoided for both anesthesia and seizure induction and a new anesthetic protocol was developed (see Results section) using 5 piglets. A previously published protocol failed to work in our hands due to lack of adequate sedation.^31^ Therefore, while subjects were on isoflurane for the initial surgical procedures, infusions of morphine (3.5 mg/kg/h) and dexmedetomidine (20μg/kg/h) were initiated and isoflurane was decreased. After the surgeries were completed, isoflurane was discontinued and piglets received chlorpromazine (2 mg/kg every 4 h). Once end-tidal isoflurane was zero for 30 min and piglets were adequately sedated (no response to noxious stimulation) without isoflurane, neuromuscular blockade was induced (rocuronium; 0.8 mg/kg) prior to induction of seizures to prevent de-instrumentation and to prevent over-breathing the ventilator during hypoventilation. After insults, breakthrough movement was treated with 0.4 mg/kg boluses of morphine and rocuronium (0.8 mg/kg), no more than once per h. To allow the drugs to metabolize, 3 h prior to recovery, dexmedetomidine infusion was discontinued and the morphine infusion was decreased to 0.5–1.4 mg/kg/h. If additional analgesia/sedation was needed to cause muscle relaxation once infusion of dexmedetomidine ceased, analgesia was supplemented with 20–40% nitrous oxide prior to recovery.

Cortical impact, mass effect, and SDH occurred as in Experimental Phase 2, but seizures were induced by local application of kainic acid (42 μg/kg) instead of with IV bicuculline. The dose of kainic acid was determined by injecting rounds of 100 μg kainic acid 15 min apart until seizures occurred, and then testing this total dose administered once in the piglets to optimize the anesthetic protocol. Kainic acid prepared at 1–1.25 mg/mL achieved more reliable and longer seizures than a solution at 5 mg/mL, possibly due to greater distribution of the larger volume when mixed in with the blood placed in the subdural space (Table 3). The kainic acid dose (an average of 0.07 μg kainic acid/g body weight among ages) is similar to the dosage of kainic acid injected into the perirhinal or occipital cortex that causes seizures in adult rodents (0.004–0.010 μg kainic acid/g body weight).^32–34^ Kainic acid was either injected 5–7 mm deep into the cortical parenchyma of the rostral gyrus leaving the needle in place for 5 min (to prevent egress) or kainic acid was mixed into the blood that was placed under the dura at one time. One piglet that received kainic acid in the cortical parenchyma developed an intraparenchymal hematoma and was excluded. In subsequent experiments, all kainic acid was mixed into the injected subdural blood to avoid intraparenchymal hemorrhage.

**Table 3.** Arterial Blood Gases and Chemistry in Experimental Phase 2

	*Pre-injury*	*Post-injury*	*Sacrifice*
Base excess (BEEcf) (mmol/L)			
Group 1	5.25 ± 0.63^a^	-2.75 ± 2.84^b^	0.00 ± 2.00^a,b^
Group 2	4.50 ± 1.32^a^	2.25 ± 1.31^b^	1.67 ± 0.67^a,b^
Group 3	4.00 ± 0.84^a^	-5.80 ± 4.33^b^	-3.75 ± 6.55^a,b^
Sham	7.00 ± 1.00^a^	3.00 ± 2.52^b^	6.00 ± 0.00^a,b^
Total CO_2_ (mmol/L)			
Group 1	29.5 ± 0.65^a^	23.7 ± 2.59^b^	27.0 ± 2.00^a,b^
Group 2	27.7 ± 1.70^a^	25.5 ± 1.26^b^	28.0 ± 1.53^a,b^
Group 3	28.8 ± 1.07^a^	22.0 ± 2.86^b^	28.0 ± 1.53^a,b^
Sham	32.5 ± 1.50^a^	28.7 ± 1.45^b^	31.0 ± 0.00^a,b^
Oxygen saturation (%)			
Group 1	99.0 ± 0.58^a^	93.2 ± 2.81^a^	94.5 ± 2.50^a,b^
Group 2	98.0 ± 0.9^a^	96.7 ± 1.11^a^	98.5 ± 1.50^a^
Group 3	63.0 ± 19.4^b*^	70.2 ± 19.6^b*^	68.8 ± 16.1^b*^
Sham	81.5 ± 4.50^a,b^	75.7 ± 6.84^a,b^	95.0 ± 0.00^a,b^
Sodium (mmol/L)			
Group 1	137 ± 0.75^a^	139 ± 0.50^b,c^	141 ± 0.88^b,c^
Group 2	137 ± 2.33^a^	141 ± 0.63^b,c^	141 ± 0.58^b,c^
Group 3	137 ± 0.75^a^	141 ± 0.89^b,c^	143 ± 1.80^b,c^
Sham	138 ± 1.00^a^	142 ± 2.96^b,c^	139 ± 0.00^b,c^
Glucose (mg/dL)			
Group 1	109 ± 14.2^a^	162 ± 3.90^b^	136 ± 17.5^a,b^
Group 2	126 ± 24.8^a^	149 ± 11.4^b^	118 ± 3.00^a,b^
Group 3	121 ± 5.79^a^	160 ± 20.4^b^	114 ± 17.1^a,b^
Sham	74.5 ± 9.5^a^	104 ± 2.89^b^	113 ± 0.00^a,b^

Data are expressed as means ± SEM. Means with different letters differ, *p* < 0.05. Group 1, *n* = 5: focal injuries + apnea, hypoventilation; Group 2, *n* = 4: focal injuries + seizures; Group 3, *n* = 7: focal injuries + apnea, hypoventilation + seizures. ^*^Treatment 1 tended to be different than treatment 3, *p* = 0.053.

BEEcf, Base excess in the extracellular fluid compartment; SEM, standard error of the mean.

Seizures were confirmed with EEG in a subset of piglets with a four-electrode montage sutured to the scalp of the piglet with two electrodes on each side amplified with an Olimex EEG-SMT device (Plovdiv, Bulgaria) recorded in BrainBay (version 1.9; open source BioSignal Software; OpenEEG project). EEGs were analyzed with dClamp (version 3.5; Staley Lab^©^ 2012). Tachycardia was a biomarker for seizure activity confirmed by EEG and was used as a surrogate for seizure duration in those piglets not instrumented with EEG. Tonic-clonic seizure activity (detected with EEG) after focal kainic acid administration was accompanied by tachycardia (> 200 bpm; Table 4), but clonic seizure activity occurred with a normal heart rate. Only seizures with tachycardia were included in “estimated seizure duration.”

**Table 4.** Kainic Acid Administration, Seizure Length, and Damage Pathology per Piglet in Experimental Phase 3

*Piglet*	*Kainic acid diluent, concentration*	*Kainic acid route*	*Length of induced seizures (min)*	*#, length of spontaneous seizures*	*Total estimated seizure time*	*% Damage contralateral hemisphere*	*% Damage ipsilateral hemisphere*
359	Water, 1.25 mg/mL	IP	180^a^	2: 15, 105 min^b^	300	22.6	64
361	Water, 1.25 mg/mL	IP (2 doses)	450^a,b^	0	450	Intraparenchymal hemorrhage, excluded	Intraparenchymal hemorrhage, excluded
365	Water, 1.25 mg/mL	SDH	210^b^	2: 60, 30 min^c^	300	12.0	50.9
369	Saline, 1.25 mg/mL	SDH	45^a^	0	45	7.2	27.0
370	Saline, 1.25 mg/mL	IP	90^b^	2: 3–5 min^c^	98	2.7	26.9
371	PBS, 5 mg/mL	SDH	30^a^	0	30	0.0	6.0
373	PBS, 5 mg/mL	IP	3^b^	3: 3–5 min^c^	12	2.5	24.5

^a^ Seizure length via EEG.

^b^ Seizure length estimated via heart rate over 200 bpm.

^c^ Seizure length estimated via visible convulsions.

EEG, electroencephalogram; IP, intraparenchymal injection of kainic acid; PBS, phosphate-buffered saline; SDH, kainic acid injected with blood to create subdural hematoma.

Apnea duration was reduced from 3 min in Experimental Phase 2 to 1 min, and oxygen was not supplemented during hypoventilation. Rounds of apnea and hypoventilation were reduced from two rounds to a single round.

Six hours after cortical impact or sham surgery, piglets were recovered from anesthesia. Sham piglets received buprenorphine (0.02 mg/kg; IM or IV) and were returned to the animal facility. Piglets receiving model injuries had impaired neurological status and stayed in the operating room where a protocol was developed for an intensive care unit (described in Results section) for recovery from anesthesia and constant observation and critical care interventions as needed.

One hour after extubation, either in the operating room/intensive care unit (piglets receiving model injuries) or in the animal facility (sham piglets), piglets underwent a neurological assessment adapted from a global asphyxia model to which assessment of focal deficits was also added (Table 5).^35^ Just prior to re-intubation at the end of the 12-h recovery period, the neurological and motor assessments were repeated.

**Table 5.** Neurological and Motor Assessment Tools

*Neurological assessment*	*Finding*	*Score*
Mental status (subtract 1 for seizures)		
	Coma, no responsiveness	1
	Stupor, responsive to vigorous stimulation only with posturing	2
	Lethargy, drowsiness or delirium	3
	Awake	4
	Subtotal	
Cranial nerves		
Pupil reflex	Unreactive (dilated and not constricting to light)	1
	Sluggish (dilated and slow to constrict to light)	2
	Normal	3
Corneal reflex	Absent	1
	Present	2
Oculovestibular reflex	Absent	1
	Present	4
Sucking reflex (7 days) Swallowing (1 month)	Absent	1
	Present	4
	Subtotal	/4 =
Reflexes		
Deep tendon reflex	Absent	1
	Hyper	2
	Normal	4
Step reflex	Absent	1
	Normal	4
Righting	Absent	1
	Normal	4
	Subtotal	/3 =
Motor: global		
Standing	Unable to stand	1
	Bears weight with abnormal posture	2
	Gets to standing with difficulty	3
	Stands normally	4
Coordination	No attempt to walk	1
	Attempts to walk but cannot	2
	Walks but fails	3
	Walks normally	4
	Subtotal	/2 =
Sensorimotor		
Withdrawal to pain	Does not respond to pain	1
	Withdraws from pain unilateral (note side)	2
	Withdraws from pain bilateral	4
Vision	No reaction, startles to face touch bilateral	1
	Startles to face touch unilateral (note side)	2
	Reacts to approaching object; does not startle to face touch	4
	Subtotal	/2 =
	**Grand Total**	
	Maximum Possible	**20**

Motor assessment	Score 0 (none), 1 (present)	Notes
Upper right		
Upper left		
Lower right		
Lower left		
All limbs equal (score = 5)		
Observe during neurological assessment: using one side more than other when walking, posturing, thrashing, righting reaction.		
		Grand total = _____/ 5

Piglets were recovered from anesthetics and extubated for a maximum of 12 h and were then re-anesthetized with isoflurane, intubated, mechanically ventilated, and transitioned to the morphine-dexmedetomidine-chlorpromazine protocol, withdrawing isoflurane. Nitrous oxide was administered if necessary to keep piglets sedated. Sham piglets were treated similarly for a similar exposure to anesthetics.

Twenty-four hours after the injury, piglets were deeply anesthetized with 3–5% isoflurane and euthanized via exsanguination by transcardial perfusion with 0.9% saline followed by 10% phosphate buffered formalin. The brain, including olfactory bulbs, and 2 cm of spinal cord were removed leaving the ventral dura intact; the brain was weighed, and post-fixed at 4°C for 5 days. Brains were weighed and photographed with and without the dura, and the presence of subdural and/or subarachnoid blood was noted. One piglet was excluded due to lack of placement of SDH. All other piglets with SDH were included in the study regardless of the area of distribution of hemorrhage in the subdural space, which varied. The total area of the SDH covering each hemisphere was determined by analyzing photographs of the separated dura and with ventral and dorsal views of the brain in Adobe Photoshop. To avoid over-estimation, any predicted superimposition of subdural blood between ventral and dura photos was only analyzed once.

The cerebellum was removed and the cerebral hemispheres divided, weighed, and coronally sliced (Fig. 3F). Blocks were paraffin embedded and stored in sealed containers at room temperature. Blocks were trimmed and 10-μm sections were made, and seven consecutive sections were mounted on poly-L-lysine-coated glass slides (Plain Micro Slides, Corning Inc., Corning, NY), dried, and stored in slide boxes at room temperature. Additional sections were made per block if needed.

One section in each block (Fig. 3F) was stained with H&E and analyzed for the tissue changes described in Experimental Phase 2, above. The volume of tissue damage and the volume of each hemisphere was estimated by averaging the area of tissue damage as a percentage of the total area of the hemisphere among all sections spanning the entire brain (10 sections per hemisphere).

### Statistical analysis

#### Physiological measures

In arterial blood, the differences in pH, partial pressure of CO_2_ and O_2_, base excess of extracellular fluid compartment, HCO_3_, total CO_2_, oxygen saturation, sodium, potassium, ionized calcium, glucose, hematocrit, hemoglobin, and lactate among time-points and among treatment groups and the interaction were tested via two-way analysis of variance (ANOVA) followed by Tukey-Kramer multiple comparisons tests. Lactate was compared pre-injury versus maximum post-injury with a Student's *t* test or over several time-points with a repeated measures ANOVA. The effect of treatment and hemisphere and the interaction were tested on damaged area via a two-way ANOVA followed by Tukey-Kramer multiple comparisons tests. The effects of treatment on left ventricle area systolic, diastolic, and fractional area change were tested with a one-way ANOVA. The difference between heart rate, end-tidal CO_2_, and oxygen saturation were compared with paired Student's *t* tests in Experimental Phase 1. The differences between heart rate, MAP, end-tidal CO_2_, and oxygen saturation minimum and/or maximum after injury versus pre-injury among treatment groups were tested via two-way ANOVA followed by Tukey-Kramer multiple comparisons tests for Experimental Phases 2 and 3. If oxygen saturation was too low to read, then 20 was inserted as a value as the lowest values successfully detected with our pulse oximetry unit were 18–22%. In Experimental Phase 2, if piglets died before receiving their assigned treatment, they were re-assigned to the treatment group matching the actual insults received.

#### Neurological scores, damage volumes, and effect of estimated seizure duration

In Experimental Phase 3, the difference in motor and neurological scores in injured versus sham piglets 8 h after injury and 20 h after injury was tested via a two-way ANOVA followed by Tukey-Kramer multiple comparison tests. The difference between the percentage of the hemisphere covered by SDH in ipsilateral versus contralateral hemisphere was tested with a paired Student's *t* test. The potential correlation between the percentage of the hemisphere covered by SDH and percentage of hemisphere damaged and the potential correlation between estimated seizure duration and percentage of damaged hemisphere were tested via Pearson correlation and the regression line plotted in SigmaPlot^®^ version 11.0 (Systat Software, Inc., San Jose, CA).

All values were expressed as means ± standard error of the mean (SEM); *p*-values <0.05 were considered significant. Statistical analyses were performed using Prism^®^ version 7.02 (GraphPad, San Diego, CA).

## Results

### Experimental Phase 1

The purpose of Experimental Phase 1 was to test epileptic agents and seizure-permissive anesthetics, SDH placement, and combinations of injuries and insults in an escalating paradigm in piglets to cause seizures and survivable acute metabolic crisis to initiate development of a model of HH (Fig. 1A). Using methohexital, an anesthetic agent used during electroconvulsive therapy in humans,^36^ we determined that bicuculline (2.5–5 mg/kg; IV), but not pentyltetrazine (PTZ; 50–150 mg/kg; IV) or penicillin applied to the surface of the cortex induced electrographic status in PND 7 piglets (Fig. 1B).^37^ PTZ induced spikes over the bilateral central area of moderate amplitude (4–6 Hz) and increased low amplitude fast activity over the bilateral frontal and central regions, but were arrhythmic lacking an electrographic seizure pattern and returned to baseline patterns within 15 min of the final dose. IV bicuculline rapidly induced generalized seizures within 10 sec in all subjects (PND 7 and 21; Fig. 1C).

Piglets (*n* = 4; PND 21) received an escalating protocol of insults and injuries (Table 1). Seizures induced by IV bicuculline lasted an average of 107 ± 29.2 min. One piglet had an additional spontaneous seizure lasting 180 min after the initial bicuculline-induced seizure ceased. The total average estimated seizure duration was 178 ± 78.7 min among piglets. As expected, apnea caused a transient decline of oxygen saturation, MAP, and heart rate (Table 6). After introduction of seizures, heart rate increased and blood pressure became undetectable via cuff measurement (Table 6). In one subject, arterial blood analysis was performed comparing pre-injury and post-injury/insults and demonstrated metabolic acidosis (bicarbonate = 25.5 vs. 17.5 nmol/L; pH = 7.43 vs. 7.32; base excess = 1 vs. −9 mmol/L) and hyperglycemia (108 vs. 239 mg/dL). Because of the low oxygen saturation and failed detection of blood pressure via a blood-pressure cuff, echocardiography was performed in two subjects before and after injuries/insults and cardiac contractile function was impaired with reduced fractional area change (Fig. 1D). One piglet died before the end of the experiment due to cardiac failure and failed resuscitation. This piglet received the maximum volume of subdural volume, mass effect, length of apnea, and hypoventilation (Table 1).

**Table 6.** Effect of Injury on Physiological Measures in Experimental Phase 1

	*Pre-injury period (mean ± SEM)*	*Post-injury (max. or min.; mean ± SEM)*
Heart rate (maximum)	155 ± 16.5	277.0 ± 16.3^*^ bpm (elevated for 178 ± 78.7 min)
Mean arterial pressure (minimum)	56.9 ± 17.5	Not detectable with cuff
End-tidal CO_2_ (maximum)	44.1 ± 4.1	70.5 ± 10.6^*^
SpO_2_ (minimum)	98.5 ± 1.5	26.5 ± 4.5^*^

^*^ Means ± SEMs differ post-injury from pre-injury, *p* < 0.05.

SEM, standard error of the mean.

### Experimental Phase 2

The purpose of Experimental Phase 2 was to determine brain injury patterns resulting from various combinations of the insults and injuries developed in Experimental Phase 1. Piglets received all the injuries (cortical impact, SDH, and mass effect) and different combinations of insults: apnea and hypoventilation alone (Group 1), seizures alone (Group 2), or apnea, hypoventilation, and seizures (Group 3, Fig. 2A). Four of 20 piglets (20%) receiving injuries and insult combinations died before the end of the experiment due to cardiac arrest following apnea.

The injuries and insults caused brief, acute, physiological and metabolic crisis. MAP increased over time (main effect of time; *p* < 0.0001) increasing after seizures and decreasing during apnea and hypoventilation in treatment groups receiving those insults (*p* < 0.05, Fig. 2B, Table 7). The increase in MAP after seizures was greater in Group 3 than Group 2, indicating that apnea and hypoventilation potentiated the effect of seizures on MAP (*p* < 0.001). The changes in MAP resolved and were not different from normal by 1 h after insults (Fig. 2C). As expected, maximum end-tidal CO_2_ increased in piglets in groups receiving hypoventilation (*p* = 0.01, Table 7). Oxygen saturation was acutely lower in all groups receiving injuries compared with pre-injury and was lower in groups with apnea and hypoventilation (Groups 1 and 3) than the seizure only group (Group 2, Table 7). In contrast to Experimental Phase 1, the ejection fraction of the left ventricle was not different as measured by a subset of piglets (*n* = 5) where echocardiography was performed (baseline ejection fraction: 46.6 ± 3.8% vs. post-injuries and insults ejection fraction: 50.7 ± 3.2%).

**Table 7.** Effect of Acute Injury on Physiological Measures in Experimental Phase 2

	*15-min pre-injury time-point*	*Post-injury (minimum or maximum)*
Mean arterial pressure minimum (mmHg)		
Group 1	51.7 ± 1.9^a^	25.5 ± 3.4^b^
Group 2	49.0 ± 2.1^a^	45.4 ± 1.8^a,c^
Group 3	46.8 ± 3.5^a^	24.3 ± 4.7^b,d^
Sham	43.5 ± 8.3^a^	30.0 ± 3.2^a,b,d,e^
Mean arterial pressure maximum (mmHg)		
Group 1	51.7 ± 1.9 ^a^	84.2 ± 10.6^b^
Group 2	49.0 ± 2.1^a^	112 ± 4.2^b,c^
Group 3	46.8 ± 3.5^a^	143 ± 12^d^
Sham	43.5 ± 8.3^a^	69.8 ± 5.2^a,b^
End-tidal CO_2_ maximum (mmHg)		
Group 1	39.0 ± 1.2^a^	66.0 ± 3.8^c^
Group 2	39.0 ± 3.3^a^	58.3 ± 4.8^b^
Group 3	42.8 ± 4.9^a^	70.5 ± 6.6^c^
Sham	35.6 ± 2.5^a^	42.8 ± 1.3^a,b^
Oxygen saturation (%) minimum		
Group 1	96.3 ± 0.6^a,c^	34.5 ± 5.6^b^
Group 2	97.6 ± 0.5^a^	83.2 ± 5.1^c,e^
Group 3	96.6 ± 0.8^a,c^	23.6 ± 2.4^b,d^
Sham	97.4 ± 0.4^a^	96.2 ± 1.1^a,e^

Data are expressed as means ± SEM. Means with different letters differ, *p* < 0.05.

SEM, standard error of the mean.

Venous or arterial blood was assessed before injury, after injury, and at sacrifice in all treatment groups and sham piglets. Base excess in the extracellular fluid compartment (BEEcf) and total CO_2_ both decreased after injury or sham surgery and remained low in all groups (Table 3). Conversely, sodium and glucose increased from pre-injury to post-injury or sham surgery and remained elevated until sacrifice in all groups (Table 3). Partial pressure of oxygen, pH, partial pressure of CO_2_, bicarbonate, potassium, ionized calcium, hematocrit, and hemoglobin did not differ between time-points or treatment groups (data not shown). Lactate was measured in a subset of piglets in Group 1 (*n* = 3) and peak lactate (4–12 h post-injury) tended to be greater than pre-injury (5.30 ± 1.33 vs. 1.63 ± 0.18, *p* = 0.052).

During the development of the model it was discovered that if subdural blood was injected after the cortical impact and mass effect balloon inflation, subdural placement increased from 38 to 90% and resulted in a discrete, thin, coalescent clot located primarily in the subdural space (Fig. 2B). This was thought to result from traumatic separation of the dural border cells, leading to a more defined subdural compartment. Failed placement of subdural blood usually resulted in the deposition of the blood in the subarachnoid space only. Most piglets with subdural blood also had a slight amount of subarachnoid blood, similar to children with AHT injuries. In a subset of piglets with model injuries (*n* = 3), the eyes were examined histologically and no optic nerve sheath or retinal hemorrhages were observed.

After the various combinations of insults, all injured groups demonstrated widespread red neurons throughout the brain (Fig. 2D) with equivalent areas of red neurons ipsilateral versus contralateral to the focal blunt injuries (Fig. 2D,E). There was no indication of edema demonstrated by vacuolization around blood vessels, and spongiosis of the neuropil in any piglet (Fig. 2D). In injured groups, focal tissue necrosis with extravasation of erythrocytes was present at the contusion site. The total area of tissue damage was not different in the hemisphere ipsilateral versus contralateral to blunt impact (main effect of hemisphere; *p* = 0.13; Fig. 2F); however, it was greater in injured piglets with apnea and hypoventilation (Group 1; *p* = 0.013) and injured piglets with seizures (Group 2; *p* = 0.02) than sham piglets (main effect of treatment; *p* = 0.003; Fig. 2F). Piglets receiving injuries, apnea and hypoventilation, and seizures did not have a larger area of tissue damage than sham piglets (Group 3; Fig. 2F) potentially due to the semi-quantitative analysis technique employed in Experimental Phase 2 as lateralization of damage was not observed. Eight piglets failed to have an SDH successfully placed and had a subarachnoid hemorrhage (usually bilateral) instead. In piglets with necropsy-confirmed SDH, the effect of SDH on the area of tissue damage in the cerebral parenchyma was tested, and this area was not different in the hemisphere ipsilateral versus contralateral to SDH (*p* = 0.75; Fig. 2G). Although metabolic crisis was induced and 20% of piglets died from cardiac arrest during the insults, the brain injury pattern was not specific to the injured hemisphere and thus, work continued to induce unilateral brain injury in Experimental Phase 3.

### Experimental Phase 3

#### Protocol adjustments

The injury pattern observed after Experimental Phase 2 was bilateral, diffuse, and not restricted to the hemisphere with the SDH. We therefore altered the study protocol to reduce apnea, place the convulsive agent focally, and extend survival to 24 h to allow greater time for evolution of the pathophysiology (Fig. 3A). Apnea and hypoventilation was reduced from two to one round and apnea was shortened from 3 min to 1 min. We further refined the anesthetic and seizure protocols to mostly avoid the GABA_A_ receptor for future work to test the effect of age on the possible GABA-related age-dependent pathophysiology of this model.^38,39^ To facilitate generation of spontaneous seizures, we recovered piglets from anesthesia overnight, which required the development of a critical care unit to monitor the animals for the extended survival period of 24 h.

### Creation of GABA-independent seizure-permissive anesthetic protocol

We tested non-GABA_A_ acting anesthetics that had previously been reported to cause sufficient anesthesia and muscle relaxation in a series of four piglets. After the surgery to create the injuries, piglets were weaned off isoflurane and a bolus of fentanyl (60–100 μg/kg) followed by a continuous infusion (30–100 μg/kg/h) that was previously reported to induce anesthesia failed to produce adequate sedation alone^40^ or in combination with dexmedetomidine (10–30 μg/kg/h).^31^ Additional boluses of fentanyl (20 μg/kg) failed to prevent movement, indicating inadequate levels of anesthesia. Dexmedetomidine was increased to a maximum of 40 μg/kg/h, which, in combination with fentanyl, induced adequate sedation, but consistently induced sinus tachycardia and induced a T wave inversion once. Therefore, alternative sedation strategies were tested using a scheme similar to neuroleptanalgesia, which uses an opioid and an antipsychotic/dopamine receptor antagonist. We tested high doses of morphine (3.5 mg/kg/h), which acts on multiple opioid receptors, in combination with chlorpromazine (1–2 mg/kg every 4 h), which was more readily available than droperidol. After the surgery, morphine in combination with dexmedetomidine (10–20μg/kg/h) with chlorpromazine provided 4–5 h of sedation during the insults. Breakthrough movement was treated with boluses of morphine (0.4 mg/kg). As an alternative to chlorpromazine, diphenhydramine (up to 2 mg/kg administered over 30 min, IV) was also tested for its sedative properties acting on the H1 receptor, but did not augment sedation. To allow the drugs to metabolize and allow for recovery from anesthesia, 3 h prior to the target time to anesthetic recovery, dexmedetomidine infusion was discontinued and the morphine infusion was reduced to 0.5–1.4 mg/kg/h. If additional sedation was needed once infusion of dexmedetomidine ceased, IV morphine was supplemented with 20–40% nitrous oxide with oxygen.

### Creation of piglet intensive care unit

To allow generation of spontaneous seizures and evolution of the pathophysiology for 24 h, recovery of piglets from anesthesia and extubation was attempted 6 h after injury. A piglet intensive care unit was created to support piglets due to the severity of the injury and resulting impaired neurological status. If the piglet's neurological status was sufficient to support spontaneous breathing and maintenance of an adequate spontaneous airway, the piglet was extubated. If the neurological status was impaired to the point that continued ventilator or airway support was necessary, then piglets were kept intubated, mechanically ventilated, and on an infusion of morphine (1.4 mg/kg/h) sufficient for sedation to maintain comfort with intubation. In this series, all piglets eventually achieved sufficient neurological status to be extubated and were moved to a cage in the intensive care/operating room for continuous observation and the arterial line left in place and blood collected every 2 h for measurement of CO_2_, pH, HCO_3_, and glucose. If pH was <7.35, HCO_3_ was >28 mmol/L, and CO_2_ was >50 mm Hg, then piglets were re-intubated. No piglets were re-intubated due to high arterial CO_2_, but two piglets were re-intubated after their breathing appeared to be labored or after periods of spontaneous apnea were observed. One piglet had a transient hypoxic episode where supplemental oxygen supplied via nose cone was sufficient to restore normal oxygen saturation and the piglet was not re-intubated. If glucose was <70 mg/dL, then 50% dextrose (0.5–1 mg/kg) was administered. If piglets were too active to safely leave the arterial and venous lines in, the arterial line was capped and secured, the venous line supplying morphine and saline was capped and secured, and buprenorphine was administered for analgesia (0.01 mg/kg, every 4 h).

### Seizures, physiology, and blood gases

Seizures induced by kainic acid were generalized tonic-clonic seizures transitioning to clonic seizures over time as determined by EEG (Fig. 3B). Tachycardia (> 200 bpm) accompanied tonic-clonic seizures and was used as a biomarker for seizures when EEG monitoring was not used. Approximately half of the injured piglets had paroxysmal motor events overnight following the induced seizures and the duration of these events were added to the “total estimated seizure duration” (Table 4).

MAP acutely changed during apnea and hypoventilation in piglets receiving model injuries, but returned to pre-injury values within 1 h after apnea and hypoventilation (Fig. 3C), similar to Experimental Phase 2 (Fig. 2B). The maximum MAP increased similarly in piglets with sham or model injuries. A rise of MAP in both sham and injured piglets was concomitant with dexmedetomidine administration (sham: pre 54.5 ± 2.5 vs. post 93.5 ± 2.5, *p* = 0.023; model injuries: pre 47.8 ± 3.4 vs. post 93.5 ± 2.5, *p* = 0.007) and may explain why sham piglets in Experimental Phase 3 had a numerically higher MAP than sham piglets in Experimental Phase 2. The minimum MAP decreased in piglets with model injuries, but not in sham piglets (Table 9). Hypercapnia was not achieved during hypoventilation as in Experimental Phase 2, perhaps because of ventilating with room air only and attempting to keep pulse oximetry >80 (Tables 7, 9). As expected, minimum oxygen saturation decreased in piglets with model injuries (during apnea; *p* = 0.004), but not in sham piglets (Table 9). One piglet required epinephrine (0.005 mg/kg) for cardiac arrest after induced apnea and was successfully resuscitated.

Arterial blood was assessed at prior to injury, at 4–7 h post-injury, and at sacrifice in injured and sham piglets. In injured piglets, lactate quadrupled from pre-injury to 4–7 h post-injury, peaking at 4–7 h post-injury, then returning to baseline by sacrifice (Table 8). Lactate did not increase in sham piglets (Table 8). Sodium continually increased from pre-injury to sacrifice in injured piglets, but did not differ over time in sham piglets (Table 8). Glucose tended to increase (*p* = 0.051), doubling from pre-injury to 8–16 h post-injury in injured piglets. In injured piglets, bicarbonate and total CO_2_ decreased after injury, returning to baseline by sacrifice, whereas sham piglets did not vary with time (Table 9). In injured piglets, hematocrit and hemoglobin increased post-injury, with maximum values at 4–7 h post-injury, returning to baseline by sacrifice, likely reflecting overall fluid status (Table 8); hematocrit and hemoglobin remained constant in sham piglets (Table 8). Arterial pH decreased in injured piglets, but not sham piglets (Table 8). Partial pressure of CO_2_ increased in both sham and injured piglets (Table 8). Partial pressure of oxygen, base excess in the extracellular fluid compartment, oxygen saturation, potassium, and ionized calcium did not differ among time-points or between treatment groups (data not shown).

**Table 8.** Arterial Blood Gases and Chemistry in Experimental Phase 3 of Model Development

	*Pre-injury*	*4–7 h post-injury*	*8–16 h post-injury*	*Sacrifice*
pH				
Injured	7.52 ± 0.03^a^	7.39 ± 0.03^b^	7.43 ± 0.02	7.43 ± 0.02^a,b^
Sham	7.57 ± 0.037^a,b^	7.53 ± 0.03^a,b^		7.4 ± 0.06^a,b^
Partial pressure of carbon dioxide (mm Hg)				
Injured	33.6 ± 2.01^a^	34.9 ± 3.01^a,b^	35.7 ± 4.47	42.8 ± 2.50^b^
Sham	32.8 ± 2.25^a^	33.5 ± 1.1^a^		53.2 ± 5.6^b^
Bicarbonate (mmol/L)				
Injured	27.4 ± 0.89^a^	22.56 ± 2.59^b^	23.7 ± 2.51	28.6 ± 0.67^a^
Sham	29.85 ± 0.55^a,b^	28.25 ± 0.95^a,b^		32.6 ± 0.6^a,b^
Total carbon dioxide (mmol/L)				
Injured	28.43 ± 0.896^a^	23.7 ± 2.68^b^	24.8 ± 2.56	30 ± 0.76^a^
Sham	30.5 ± 0.5^a,b^	29 ± 1^a,b^		34.5 ± 0.5^a,b^
Sodium (mmol/L)				
Injured	137.1 ± 0.738^a^	138.7 ± 1.063^a^	144.3 ± 1.93^*^	146.4 ± 1.73^b^
Sham	135.5 ± 0.5	139 ± 2		137 ± 1
Glucose (mg/dL)				
Injured	102.7 ± 10.32	181.1 ± 35.04	223 ± 45.44^†^	153.57 ± 15.25
Sham	108 ± 22	82 ± 21		211.5 ± 42.5
Hematocrit % (PCU)				
Injured	21.71 ± 1.17^a^	27.57 ± 1.325^b^	20.83 ± 2.12	16.86 ± 2.849^a^
Sham	22.5 ± 1.5^a,b^	29.5 ± 0.5^a,b^		21.5 ± 2.5^a,b^
Hemoglobin (g/dL)				
Injured	7.371 ± 0.398^a^	9.41 ± 0.436^b^	7.017 ± 0.696	5.743 ± 0.971^a^
Sham	7.65 ± 0.55^a,b^	10.05 ± 0.15^a,b^		7.35 ± 0.85^a,b^
Lactate (mmol/L)				
Injured	1.1 ± 0.084	4.823 ± 1.513^‡^	3.027 ± 0.916	0.853 ± 0.125^§^
Sham	0.7 ± 0.4	0.83 ± 0.23		1.015 ± 0.215

Data are expressed as means ± SEM. Means with different letters differ via two-way repeated measure with Tukey-Kramer's post hoc test, *p* < 0.05.

^*^ 8–16 h post-injury differs from pre-injury in injured piglets via one-way analysis of variance (ANOVA).

^†^ 8–16 h post-injury tended to be different than pre-injury in injured piglets via one-way ANOVA, *p* = 0.0515.

^‡^ 4–7 h post-injury differs from pre-injury in injured piglets via one-way ANOVA.

^§^ Sacrifice differs from 4–7 h post-injury in injured piglets via one-way ANOVA.

SEM, standard error of the mean.

**Table 9.** Acute Effect of Injury on Physiological Measures in Experimental Phase 3

	*Pre-injury*	*Post-injury (minimum or maximum)*
Mean arterial pressure minimum (mmHg)		
Model injuries	58.7 ± 3.3^a^	21.6 ± 5.7^b^
Sham	62.1 ± 7.9^a^	45.5 ± 5.5^a,b^
Mean arterial pressure maximum (mmHg)		
Model injuries	58.7 ± 3.3^a^	109.8 ± 10.9^b^
Sham	62.1 ± 7.9^a^	115.5 ± 3.5^b^
End-tidal CO_2_ maximum (mmHg)		
Model injuries	34.4 ± 2.1^a^	53.0 ± 0.0^b^
Sham	34.25 ± 2.3^a^	56.6 ± 1.9^b^
Oxygen saturation (%) minimum		
Model injuries	99.45 ± 0.5^a^	67.6 ± 7.7^b^
Sham	100 ± 0^a^	97.5 ± 0.5^a^

Data are expressed as means ± SEM. Means with different letters differ, *p* < 0.05.

SEM, standard error of the mean.

### Neurological status

Upon recovery from anesthesia, piglets receiving model injuries had lower neurological and motor scores in the evening (8 h post-injury) and the following morning (20 h post-injury) compared with sham piglets (Fig. 3D, *p* < 0.05). Motor scores were lower at 20 h post-injury in piglets receiving model injuries versus sham piglets (Fig. 3E, *p* < 0.05). Most piglets receiving model injuries were extubated and laid quietly on their side in the recovery cage, appearing to be comfortable and breathing well. Piglets had their eyes open or closed for various periods during the 12-h extubated period. Most piglets were hypoactive, were unable to bear weight with the rear legs, and had variable asymmetry in the front legs. Two piglets were re-intubated before the scheduled re-intubation: one piglet was spontaneously apneic and one had labored breathing. One piglet had an emergency re-intubation after poor spontaneous respiration. One piglet required glucose for hypoglycemia. Sham piglets were ambulatory and were returned to the animal facility overnight.

### Brain pathology

**FIG. 4.**
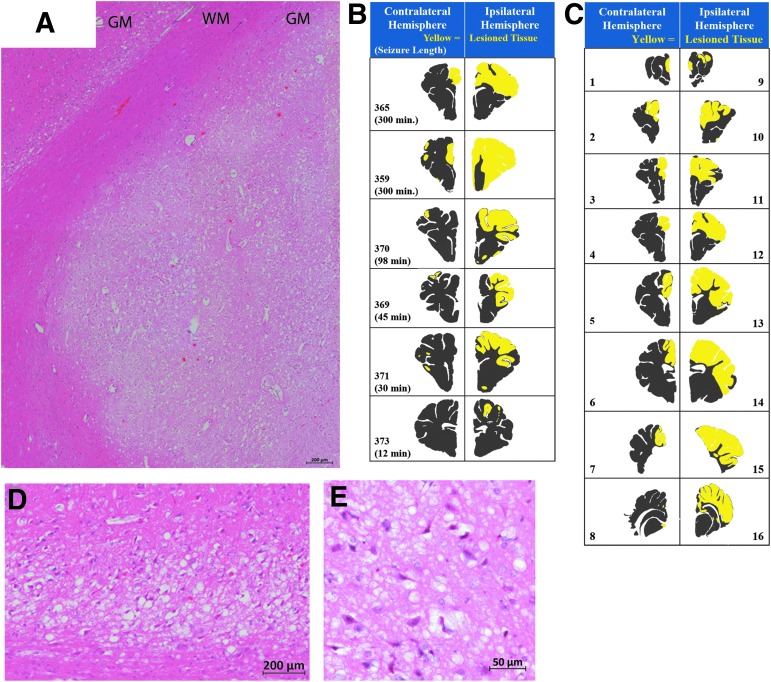
Patterns of brain pathology from Experimental Phase 3. **(A, D, E)** H&E-stained sections showing acute hypoxic-ischemic type injury of cortex with red neurons. (A) Stitch of H&E stained showing damaged gray matter (GM) adjacent to normal appearing white matter (WM). **(D, E)** Photomicrographs at higher magnification demonstrating red neurons and vacuolization of neurons and neuropil. **(D)** Representative sections of each injured piglet depicting the area of microscopic tissue damage ipsilateral or contralateral to the focal impact (SDH, cortical impact, mass effect; yellow: damaged tissue, gray: normal tissue). **(B, C)** Maps demonstrating the hemispheric microscopic tissue damage of one piglet (365) rostral to caudal (section number corresponds to Fig. 3F). H&E, hematoxylin and eosin.

Piglets that received model injuries had widespread cortical changes consisting of red neurons, patchy rarefaction and pallor of cortical neuropil, and variable perivascular vacuolization in the hemisphere ipsilateral to the SDH (Fig. 4A,D,E). These acute hypoxic-ischemic-type changes were extended continuously throughout the cortical ribbon straddling several gyri in some piglets, were discontinuous, multi-focal throughout the affected hemisphere in other piglets, and extended beyond the region of cortical impact (Fig. 4C). Piglets with the largest percentage of damaged cortex also exhibited a patchy pallor of cortical white matter. Acute hypoxic-ischemic type change was additionally present in the basal ganglia in the most severely affected piglets (Fig. 4B). The thalamus was usually spared although the most severely injured piglet had a small focus of damage.

Piglets receiving model injuries had greater injury to the hemisphere ipsilateral versus contralateral to focal impact (33.2 ± 8.5% vs. 7.8 ± 3.4%, *p* = 0.044) and sham piglets (Fig. 3G). Purkinje red cell change was observed, but was not different among shams versus model injuries (sham contralateral: 5.1 ± 3.06, sham ipsilateral: 0.64 ± 0.17, model injuries contralateral: 7.87 ± 6.7, model injuries ipsilateral: 1.06 ± 0.71). Among injured piglets, the estimated duration of seizure (induced + spontaneous) was positively correlated with the percentage of ipsilateral cerebral hemispheric cortex that was damaged (r = 0.913, *p* = 0.011; Fig. 3H) as well as the contralateral hemisphere (r = 0.862, *p* = 0.027; Fig. 3I). Neither the length of the seizure nor the percentage of the ipsilateral hemisphere damaged differed among piglets with kainic acid administered in the brain parenchyma versus administered mixed with the subdural blood, but one piglet that received kainic acid in the parenchyma had a large intraparenchymal hemorrhage within the cortical ribbon and was excluded; kainic acid was introduced with the SDH blood thereafter (Table 4). Within piglets with model injuries, the area of the hemisphere covered by SDH was greater in the ipsilateral versus contralateral hemisphere (Fig. 3J, *p* = 0.002); among all hemispheres, SDH area positively correlated with area of tissue damage (*p* = 0.021, Fig. 3K). Of note, because the blood was injected into the subdural space along the medial aspect of the frontal convexity, some subjects had a small amount of subdural blood that extended to the medial surface of the contralateral hemisphere. This may be reflected in a small amount of focal damage in this region of the contralateral hemisphere in some subjects (Fig. 4B,C).

Brain weights at 24 h did not differ in sham versus piglets receiving injuries (57.8 ± 2.6 vs. 61.6 ± 2.4, *p* = 0.47). In animals receiving model injuries, hemispheric weight did not differ ipsilateral versus contralateral to the site of focal impact (23.81 ± 0.91 vs. 22.44 ± 0.60 g, *p* = 0.22).

**FIG. 5.**
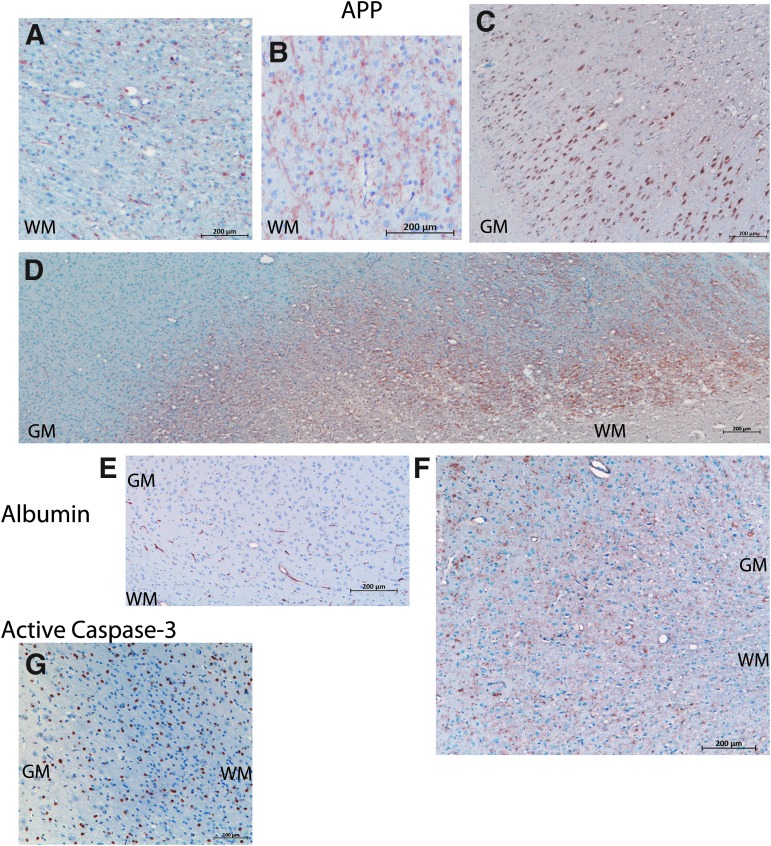
Brain pathology from Experimental Phase 3. **(A,B)** APP in the white matter (WM). (A) Axonal spheroids positive for APP (brown) in the WM of the cortex adjacent to the gyri that underwent cortical impact. (B) APP-positive neurites revealing additional WM injury that appeared normal via H&E. **(C)** APP-positive neurons in the gray matter (GM). **(D)** A low power stitch of photomicrographs demonstrating a geographic pattern of APP in the internal capsule (WM) and adjacent GM observed in one piglet (359). **(E)** Albumin (brown) in sham piglets was restricted to blood vessels. **(F)** In injured cortex, albumin was extravasated from blood vessels. **(G)** Active caspase-3 (brown) was widespread in sham piglets, and was not specific to damage in this age of piglet. H&E, hematoxylin and eosin.

Immunohistochemistry for APP often overlapped with the damage on H&E staining and also revealed additional injury in the white matter (Fig. 5A–D). Traumatic axonal injury was observed (Fig. 5A,B). Adjacent to damaged areas, but distant from the lesion from cortical impact, neurons exhibited cytoplasmic positivity for APP (Fig. 5C). In the piglet with the most extensive damage (359, Fig. 4D), large geographic zones of positivity for APP were observed (Fig. 5D). All piglets with model injuries had evidence for vasogenic edema as determined by extravasation of albumin around blood vessels in areas with red cell change (Fig. 4F).

Active caspase-3 was expressed by many non-pyramidal neurons or glial cells in both sham piglets and piglets receiving model injuries (Fig. 5G, sham piglet).

## Discussion

This series of investigations has produced a model that enables the creation of hemispheric damage largely restricted to the hemisphere underlying the SDH in a pattern similar to children with radiographical unilateral hemispheric hypodensity.^6,7^ The tissue damage extends beyond the cortical impact site and the area covered by the unilateral SDH, and encompasses the entire cerebral cortex with relative sparing of deep nuclei, apart from in the most severe cases. This multi-injury model includes cortical impact, mass effect, seizures, apnea, and hypoventilation as well as secondary metabolic acidosis. The cortical ribbon displayed extensive red cell change and blood–brain barrier breakdown. Damage was restricted to the ipsilateral hemisphere when the epileptic agent was placed focally, the anesthetic and epileptic agents were non-GABAergic, and piglets were supported for 24 h after injury. The extent of red cell change positively correlated with SDH area and estimated seizure duration. Piglets recovered from anesthesia were neurologically impaired similar to children with this injury pattern. It is difficult to tell if this reduced neurological status is permanent or due to the potential temporary suppression of neurological function in a post-ictal state, but we hypothesize that the hemiparesis is likely permanent.

In this set of experiments, we exposed piglets to different combinations and durations of insults in three successive phases (Experimental Phases 1, 2, and 3). The second experimental phase failed to cause unilateral hemispheric hypodensity, only causing a bilateral diffuse pattern. In the third experimental phase, a widespread acute hypoxic-ischemic type injury pattern primarily restricted to one hemisphere was achieved. In Experimental Phase 2, with two rounds of long apnea (3 min), mass effect, and systemic administration of convulsant, the pattern of bilateral acute hypoxic-ischemic type injury indicated primarily anoxic damage. In contrast, the pattern in Experimental Phase 3 encompassed the entire cortical ribbon or large patches of the cortex primarily restricted to the ipsilateral cerebral hemisphere when apnea was reduced (1 min) and when seizures were induced focally. The unilateral damage we observed in Experimental Phase 3 is not explained solely by global anoxia, as the contralateral hemisphere was relatively unaffected. Although not evaluated in this model, the unilateral pattern of HH is unlikely to result from neck injury and/or nerve reflexes from the dura, which cause global apnea, a mechanism purported by Geddes and colleagues and others.^10,11,41–43^ An alternate possible mechanism is a regional metabolic mismatch between supply and demand. The exact pathophysiological mechanisms by which widespread unilateral hemispheric hypoxic-ischemic injury is initiated and propagated in children are not fully understood, but similar findings were produced in this model in the absence of angular acceleration/deceleration. In this model, the tissue damage covered multiple arterial perfusion territories without evidence of arterial occlusion, venous thrombosis, or watershed infarct. One potential mechanism for this pattern of injury results from an irrecoverable imbalance between metabolic demand and substrate delivery that overwhelms the compensatory mechanisms available to the immature brain. This mismatch can affect the entire brain, or can preferentially affect one hemisphere over the other. The specific physiological events resulting from the various types of insults in children with severe AHT and reproduced in this model may lead to increased metabolic demand (seizures, or seizure-associated neurophysiological stressors) in the setting of reduced cerebral blood flow (cortical impact, SDH, mass effect) resulting from both arteriole vasoconstriction and decreased global blood flow, and further exacerbated by hypoxia and hypercarbia induced by apnea and hypoventilation.

Animal models of shaking have not resulted in the HH pattern observed in our model. Infant mouse pups were shaken (12 min, 6-sec rounds shaking, 6-sec rest) for 3 consecutive days starting on day 6 after birth in a hypoxic chamber (8% O_2_).^19^ Rates of angular acceleration and deceleration for the small rodent brain were not calculated and mice were anesthetized, injured, and recovered without airway protection or oxygen saturation monitoring.^19^ Brain damage was measured via brain weight of both hemispheres at 7 or 14 days after injury, “cortical hemorrhage” was quantified, but there was no specification of SDH or subarachnoid hemorrhage, and no histopathology was reported.^19^ It is difficult to know what type of damage was achieved with no microscopic images, and is unlikely to be unilateral. In a similar experiment using a gyrencephalic model, lambs were manually shaken, but angular acceleration/deceleration and velocities were not measured.^20^ These lambs were ventilated so were not hypoxic, but the smallest lambs died unexpectedly. Shaken lambs that died had focal areas of albumin extravasation and APP-positive neurons, but a hypoxic-ischemic injury pattern was absent and focal SDH, when present, was very small.^20^ Finally, in another report, piglets that received cyclic head rotations with kinematics carefully scaled to the human infant brain had modest damage with only 0.1% of the brain injured with diffuse red neurons and axonal damage compared with 30% of the hemisphere in our model and with no evidence of widespread hypoxic ischemic pathological pattern.^21^ Cyclic shaking caused extra-axial hemorrhage covering only 2% of the brain surface.^21^ The pattern of unilateral or bilateral HH as observed in children did not occur after shaking in these animal models. Thus, the circumstances required to cause the physiological cascades resulting in HH remain unclear, but to date have not been replicated by animal experiments involving shaking mechanisms.

The unilateral hypoxic-ischemic type injury pattern observed in Experimental Phase 3 of the present experiments occurred with less apnea, less hypoventilation and hypercarbia, and higher MAP than in Experimental Phase 2, but with potentially more localized seizures. The anesthetic protocol in Experimental Phase 3 included dexmedetomidine, which causes a transient increase in blood pressure,^44^ which may have explained the improved blood pressure. Although a series of children with AHT who developed HH more frequently required vasopressors,^3^ low blood pressure may not be a key driver of the pathophysiology.^45^ Blood gases, pH, and lactate were worse in Experimental Phase 3 than Experimental Phase 2, with less apnea and hypoventilation. The longer total estimated seizure duration may have driven the metabolic acidosis in Experimental Phase 3. The percentage of damaged hemisphere was positively correlated with estimated seizure duration. Similarly, in children with AHT, seizure severity was the single clinical feature that was positively correlated with the proportion of brain damage (restricted diffusion via magnetic resonance imaging [MRI]).^45^ Although kainic acid has been theorized to be directly neurotoxic, in piglets with brief seizures, damage did not expand beyond the site of cortical impact. We can conclude that the widespread damage was not caused by kainic acid itself. It remains to be determined if the metabolic mismatch creating the hemispheric damage is due to the combinations of injuries and insults or if severe seizures alone can induce this pathology.

Creation of a new anesthetic protocol was required in this study to adequately sedate piglets while avoiding the GABA_A_ receptor to test the age-dependent effects of injury; and will be required in future experiments.^38,39^ Anesthetic protocols using fentanyl alone or in combination of dexmedetomidine failed to induce adequate sedation as previously described in piglets.^31,40^ It is possible that these protocols failed to work in our hands because we induce neuromuscular blockade only after morphine and dexmedetomidine successfully sedated animals to ensure that sedation is adequate during paralysis. Fentanyl is a μ-opioid receptor agonist that works well in humans, but we found that morphine, which binds to multiple opioid receptors, worked better as a sedative in piglets, with a dose previously used for anesthesia in elective open heart surgery.^46^ The amount of dexmedetomidine we found necessary to induce anesthesia in piglets was 10 to 100 times greater than required in children.^44,47^ Chlorpromazine, used to treat psychotic mania, was added to the combination. After sedation was achieved, rocuronium was given to induce muscle relaxation (no more frequently than every 45 min). This combination provided at least 4 h of non-GABAergic anesthesia that was seizure-permissive.

In previous models of pediatric brain injury, we demonstrated that neither cortical impact^22,23^ nor SDH^48^ cause HH, but we have not yet tested the theory that HH could be due to seizures alone. Seizures occur in 70–80% in infants who develop HH,^5,6,9,45,49^ increasing metabolic demand potentially, synergistically contributing to damage, or perhaps, driving the injury alone; the SDH may be a result of the brain trauma without contributing to the pathophysiological cascades. In our large-animal models of TBI, we have demonstrated that cortical impact alone^22,23^ or SDH alone causes focal damage,^15^ but not the extensive widespread acute hypoxic-ischemic type damage observed in HH. In immature rodents prior to 18 days of age, parental kainic acid (1–6 mg/kg) induced severe, generalized tonic-clonic seizures without any histopathological damage, and limbic seizures and “red neurons,” neuronal dropout, and a gliotic reaction and scarring at 18 days of age and thereafter.^50,51^ It is difficult to make a direct comparison of developmental stage between rats and piglets, but these ages (day 18 in rats and 4 weeks in piglets) are just before weaning in both species and may be comparable. However, no edema, vacuolization of veins, vacuolization of neuropil, or blurring of the gray-white matter was reported at any age or any time after injury in rodents.^50^ Further, the neuronal damage observed in rodents was primarily in the limbic system and deep gray matter regions^50^ opposite to the pattern observed in our HH piglet model where we observed extensive damage of the cortical ribbon with relative sparing of the deep brain regions and hippocampus. To our knowledge the effect of kainic acid alone on brain pathology has not been determined in swine of any age. We are aware of only one report of the effect of kainic acid alone in a gyrencephalic species: ingestion of domic acid, a kainic acid analogue, in sea lions caused unilateral atrophy of hippocampi, but the remainder of the brain was not described.^52^

Another piece of evidence that severe seizures may be a critical driver of HH is the phenomenon of hemiconvulsive hemiplegia syndrome in infants and toddlers. Hemiconvulsive hemiplegia can occur in otherwise healthy children from prolonged unilateral seizures or status epilepticus resulting in unilateral hemispheric damage and hemiplegia and unilateral encephalopathy similar to HH as seen with MRI.^53^ The neuropathology of the affected hemisphere parallels our HH model with a widespread hypoxic-ischemia pathology pattern with vacuolization of the neurons and neuropil.^54^ Whereas some of these cases are associated with genetic seizure disorders (CACNA1A), they demonstrate that a severe seizure disorder can cause unilateral hemispheric encephalopathy. It seems possible that traumatic injuries that are asymmetric may be the initial trigger for seizures in the predominantly unilateral form of HH.

In conclusion, we have created the first model of HH where injuries (cortical impact, unilateral SDH, mass effect) and insults (seizures, apnea, hypoventilation) caused profound, widespread acute hypoxic-ischemic type injury throughout the cortical ribbon ipsilateral to impact and SDH, encompassing a majority of the hemisphere with relative sparing of the contralateral hemisphere and deep nuclei. Future studies will determine the effect of age on the pathophysiology of HH, determine if stopping seizures can prevent the extensive damage, and will explore the timing and the mediators of vasogenic edema. Identification of potential therapeutic targets may limit the evolution of this injury, improve innate repair efforts,^24^ and reduce the burden of disability and death from this severe injury resulting from AHT.
